# Evidence of uneven selective pressure on different subsets of the conserved human genome; implications for the significance of intronic and intergenic DNA

**DOI:** 10.1186/1471-2164-10-614

**Published:** 2009-12-16

**Authors:** Scott Davidson, Andrew Starkey, Alasdair MacKenzie

**Affiliations:** 1 School of Medical Sciences, Institute of Medical Sciences, University of Aberdeen, Foresterhill, Aberdeen, AB25 2ZD, UK; 2 School of Engineering, Fraser Noble Building, Kings College, University of Aberdeen, Aberdeen, AB23 UE4 , UK

## Abstract

**Background:**

Human genetic variation produces the wide range of phenotypic differences that make us individual. However, little is known about the distribution of variation in the most conserved functional regions of the human genome. We examined whether different subsets of the conserved human genome have been subjected to similar levels of selective constraint within the human population. We used set theory and high performance computing to carry out an analysis of the density of Single Nucleotide Polymorphisms (SNPs) within the evolutionary conserved human genome, at three different selective stringencies, intersected with exonic, intronic and intergenic coordinates.

**Results:**

We demonstrate that SNP density across the genome is significantly reduced in conserved human sequences. Unexpectedly, we further demonstrate that, despite being conserved to the same degree, SNP density differs significantly between conserved subsets. Thus, both the conserved exonic and intronic genomes contain a significantly reduced density of SNPs compared to the conserved intergenic component. Furthermore the intronic and exonic subsets contain almost identical densities of SNPs indicating that they have been constrained to the same degree.

**Conclusion:**

Our findings suggest the presence of a selective linkage between the exonic and intronic subsets and ascribes increased significance to the role of introns in human health. In addition, the identification of increased plasticity within the conserved intergenic subset suggests an important role for this subset in the adaptation and diversification of the human population.

## Background

Although it is widely accepted that genome changes have driven evolution, there is still a lack of consensus as to what aspect of genome function are most affected to bring about phenotypic change. Many conjecture that changes within exonic coding regions are most important [1,2] whilst others suggest changes within regulatory regions as the driving force of adaptive and evolutionary change [3]. While the majority of changes produce no phenotypic effects a small number produce the characteristics that define individuals and populations of humans [4]. These functional polymorphisms are subject to selection by influences such as environment (climate, food availability, predation or disease) and sexual selection [5]. However, functional polymorphisms also contain a sub group of polymorphisms that reduce fitness and may increase disease susceptibility [6].

One method to address the question of where the majority of functional polymorphisms lie within the human genome is to examine the densities of polymorphisms within the different functional components of the conserved human genome. Thus, functional portions of the genome under the strongest selective pressure will contain less polymorphisms due to removal of less fit individuals from the population at an early age. The advantages of examining the conserved genome to select for functional importance is that mechanistic bias towards particular subsets is removed and the importance of a particular sequence to survival is defined by its retention through evolution. Once these conserved sequences have been identified they can be divided into functional subsets and densities of polymorphisms within these subsets can be compared. Thus, if one portion of the conserved genome contains a lesser density of polymorphisms, despite being conserved to an identical degree, it can be assumed that this portion has been subjected to a higher degree of purifying selection within the human population and is consequently more important in maintaining species fitness and conferring disease susceptibility prior to reproductive age if compromised.

In order to determine the densities of polymorphisms, in the form of SNPs [7] across the entire coding or non-coding portions of the conserved human genome we used a novel approach that allows each nucleotide base within a chromosome to be viewed as a member of a set. Using this set theory approach we were able to further group these nucleotide sets into defined subsets (exonic, intronic or intergenic) and have intersected these subsets with further definitions of the genome i.e. whether the nucleotide base is polymorphic or not. Extending this approach, comparative analysis of these subsets allows us to examine SNP density intersected with the evolutionary conserved regions (ECRs) of nine other species with the human genome at three different conservation levels, or stringencies, of conservation.

Using this simple but unique set theory approach we have been able to demonstrate that, in keeping with the current understanding of its importance in evolution and health, the conserved exonic subset has a significantly reduced density of SNPs and has therefore been subjected to greater selective pressures than other areas of the genome. Unexpectedly, comparison of the SNP densities between the intergenic and intronic components, both previously considered "junk DNA", demonstrated significant differences in SNP densities, such that the intronic portion had a statistically identical SNP density to the exonic component and the intergenic component contained a significantly higher SNP density. These observations demonstrate that the conserved intronic subset of the human genome has been subjected to identical levels of purifying selection as the exonic component within the human population. These novel and far reaching observations point to a critical role for conserved intronic sequences in the maintenance of species fitness and human health and give added weight to the analysis of intronic polymorphisms in the search for the causes of human genetic disease. In addition, the higher SNP density within the intergenic subset is indicative of its important role in driving the adaptive changes that reflect diversity within the human population.

## Methods

The genomic data of chromosomal positions for transcripts and the positions of exons within those transcripts were downloaded from the UCSC genome browser through the table browser portal [8] from the UCSC genes table [9]. Chromosomal positions of repeat elements were taken from the UCSC genome browser from the repeat-masker table [10]. SNP data for this analysis was based on the NCBI repository for SNPs; dbSNP version 129 (dbSNPv.129), that holds 12, 483,371 true SNPs. 6, 726,791 of these SNPs currently hold validated status, and 6, 406,772 of these lay within the autosomal chromosomes [11]. Coordinates of pairwise alignments to the human genome were taken from the ECR Browser through the ECRBase portal [12]. The species aligned to human were Pt; Pan troglodytes, Rm; Macaca mulatta (Rhesus Macaque), Cf; Canis familiaris, Mm; Mus musculus, Rn; Rattus novergicus, Md; Monodelphis domestica, Gg; Gallus gallus, Xt Xenopus tropicalis and Dr; Danio rerio.

These data were held on a MySQL database implemented on a 56 node High Performance Cluster (HPC) IBM blade array operated by Microsoft compute cluster server 2003. All programs were written in Visual Basic .net on the Microsoft .net 3.5 framework, in a parallel design using the database to pass messages and data to the worker nodes. The database was designed so that the queries were optimized during the analysis process, and also to optimize subsequent analysis of the results. In utilizing set theory, each chromosome was considered as a set with its members being its base pairs. Each chromosome was considered separately as an entity of DNA of independent evolutionary path. The bases of each chromosome were categorized according to their position with respect to the different annotation information gathered.

All autosomal chromosomes were analysed (2, 867,732,772 bases), although the × and Y chromosomes were removed from the analysis due to being under different selective pressures and being represented differently within the population. The mitochondrial genome was also removed from the analysis. We also removed repetitive regions (1, 288,883,792 bases) as the repetition, frequency and random nature of these repeat regions present problems when using pairwise alignment analyses. Un-sequenced regions of the genome, such as centromere regions of each chromosome were also removed from the analysis as no alignments or SNPs can be mapped to these regions (185, 443,999 bases). From the starting genomic annotations, set algebra was used to define subsets for further investigation, as described in Table 1. The use of set theory in this manner exploited the data currently available for polymorphisms (SNPs) and also the intronic, exonic and intergenic regions of the genome. The total number of bases within each region type was calculated. Using the chromosomal coordinates of the SNPs, the number of SNPs within each region type was also calculated. This allowed a basic description of SNP density within each region type to be calculated as:

**Table 1:** Set theory algebra of genomic regions from annotation data

Genomic Region	Set Algebra
Chromosome	*Chromosome\Repeats*

Transcripts	*Transcripts\Repeats*

Exons	*Exons\Repeats*

Introns	*Transcripts\Exons*

Intergenic	*Chromosome\Transcripts*

Conserved	*Alignments\Repeats*

Conserved Exonic	*Exons*∩*Alignments*

Non-Conserved Exonic	*Exons\Alignments*

Conserved Intronic	*Introns*∩*Alignments*

Non-Conserved Intronic	*Introns\Alignments*

Conserved Intergenic	*Intergenic*∩*Alignments*

Non-Conserved Intergenic	*Intergenic\Alignments*

\ denotes set difference, ∩ denotes set intersection.

The analysis was carried out on each chromosome with pairwise alignments of each species aforementioned at three different selective "stringencies" of 70%, 80% and 90% over 100 base pairs. However at the higher level of stringencies the size of conserved genome for large evolutionary depth was small and the number of SNPs reduces to a very small number. Therefore, in order to keep the analysis statistically valid across all species the 70% data was selected for the majority of the analysis in this paper although the 80% and 90% data demonstrated a similar trend. Statistical analyses of the results were carried out in MATLAB version 7.1 (Mathworks) and Microsoft Excel 2003. Tests of normality were undertaken using the Jarque-Bera test (JB test) on the mean SNP density counts for all regions as described in Table 1 for each chromosome and the null hypothesis of normality could not be rejected[13]. Thus, the average chromosomal SNP density is a fair method of representing the data across the chromosomes and allows the ANOVA statistical test to be used for comparison between the subsets at a 95% confidence level. The average validated SNP density per kilo base (kb) of the total genomic sequence, based on the most current dbSNP database (dbSNPv.129), is approximately 2.6.

## Results

### Evaluating bias within the dbSNP dataset

**Figure 1:**
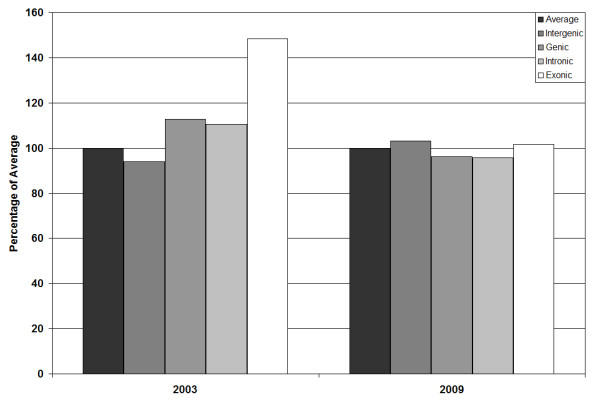
**Bar Chart displaying SNP density for 2003 study **[14], **compared to the present study (2009)**.

It has been recognised in the past that due to the methodology used in discovering SNPs within the human genome, there has been a bias within dbSNP to hold SNPs that are found within protein coding regions [14]. In undertaking an investigation of SNP density within the conserved human genome, it was necessary to understand the extent of bias within the present dbSNP database (dbSNP129). Using the figures given by Zhao, *et al*. we have compared SNP data from 2003 to that held within dbSNP in 2009 (figure 1) [14]. It can be seen that the 48% bias in exonic SNPs has been diluted to negligible levels due to the genome wide SNP discovery analysis of projects such as HAPMAP [15,16].

Confirmation of the normal distribution of SNP density across the human nuclear genome and between different chromosomes was undertaken to detect any possibility of chromosomal bias density of SNPs that might harm the validity of the ANOVA analysis to be used later in the current study. However, using a JB test we found that mean chromosomal SNP density followed a normal distribution. While we accept that other types of variation such as CNVs and tandem repeats are also important in inducing phenotypic variation and conferring disease susceptibility [17] we were unable to include these in the current analysis as there is currently insufficient information as to the distribution and density of these variants within the human population although this situation may change soon[18].

### SNP density with increasing evolutionary depth

We defined a chromosomal baseline for SNP density (2.6 per kb; based on the dbSNP 129 database), and have determined that the major reservoirs of human SNP variation within the human genome conserved at three different stringencies within amniotes are to be found within the non-coding portion of the genome (the intergenic and intronic subsets) (Table 2). For example, the conserved sequence between human and common chimpanzee has only 4.4% of all SNPs occurring within the exonic sequence region, the remaining 95.6% is within the intronic and intergenic regions (Table 2).

**Table 2:** Numbers of SNPs within the conserved human genome at evolutionary stringency of 70%

% Identity	Species	Subset					
		
		Exonic		Intronic		Intergenic	
		
		Count	% of Total	Count	% of Total	Count	% of Total
70	Pt	142762	4.4	1412208	43.3	1704439	52.3
	
	Rm	123594	4.4	1224725	43.4	1471048	52.2
	
	Cf	132676	4.5	1289667	43.5	1539598	52.0
	
	Mm	90324	14.4	263730	42.1	272453	43.5
	
	Rn	85933	15.1	236673	41.6	246717	43.3
	
	Md	59425	27.1	75357	34.4	84128	38.4
	
	Gg	34459	44.3	21368	27.5	21962	28.2
	
	Xt	18291	58.1	6935	22.0	6281	19.9
	
	Dr	9947	62.3	3145	19.7	2886	18.1

**Figure 2:**
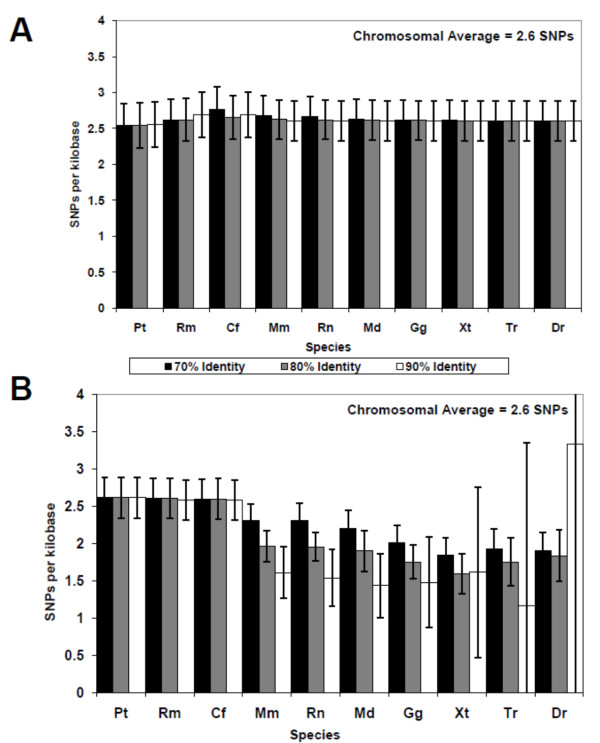
**Bar charts demonstrating SNP densities with the conserved (A) and non-conserved (B) regions of the human genome as determined by pairwise alignment with nine other species (Pt; Pan troglodytes, Rm; Rhesus Macaque, Cn; Canis familiaris, Mm; Mus musculus, Rn; Rattus novergicus, Md; Monodelphis domestica, Gg; Gallus gallus, Xt Xenopus tropicalis, Dr; Danio rerio) at three different selective stringencies (70%; black bars, 80% grey bars; 90%; white bars over 100 base pairs) showing the number of SNPs per kilobase (y-axis) plotted against species (x-axis) that increases in evolutionary "depth" from left to right**.

We sought to determine if there was a relationship between SNP density and evolutionary depth as it has been shown that highly conserved sequences in the human genome are indicative of functionally important sequences within genes [19], introns [20], and within intergenic regions [21,22]. An examination of the SNP density within non-conserved regions of the human genome shows that the density does not differ significantly from the chromosomal average (Figure 2A). Conversely, if we examine SNP densities in portions of the genome that have been conserved we are able to see a significant reduction of SNP density both with increased conserved stringencies and with evolutionary depth (Figure 2B). At a conservation stringency of 70% over 100 bp, we can see a significant reduction of SNP density in parts of the genome conserved between human and chicken or earlier. At an 80% and 90% conservation stringency we are able to see significant reductions in SNP density in parts of the genome conserved between rodents and humans. The 90% conservation stringency at the conservation between Frog - human (Xt) and Zebrafish - Human (Dr) (conservation over 350-450 million years) looses consistency (Figure 2B) because at the 90% conservation stringency over 100 base pairs, many chromosomes are excluded from the analysis as they do not contain sequences conserved to this degree. However, this does not detract from the SNP density figures provided for Opossum - Human (Md) and Chicken - Human (Gg) from the 90% conservation that show conservation up to 300 million years following the trend displayed in the 70 and 80% conservation stringencies.

The results of this analysis demonstrated that SNPs occur at a significantly lower rate in the most highly conserved regions, conserved since the common ancestors of humans and birds, amphibians and fish, as confirmed by one-way ANOVA analysis [23]. The observation that more highly conserved sequences are less polymorphic is consistent with the hypothesis that evolutionary conservation highlights functionally important sequences. Thus, genetic lesions within these regions may reduce species fitness prior to, and during, reproductive age. As a result, these genetic lesions will not be so likely to be seen as polymorphisms within the human population. Conversely, polymorphisms within regions that are less functionally important are less likely to reduce species fitness in ability to reproduce and as a result can be maintained within the population.

### Comparisons of SNP density between conserved subsets

**Figure 3:**
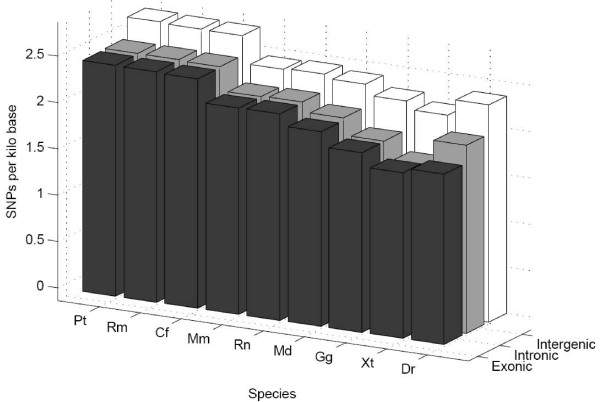
**Bar chart demonstrating mean SNP densities within different subsets of the conserved genome as determined using pairwise analysis at 70% conservation with nine different species**. X-axis shows increasing evolutionary depth.

Because they had been conserved to the same degree we initially hypothesised that SNPs would occur with equal density throughout the conserved genome, irrespective of the identity of the conserved subset. We carried out ANOVA analysis to determine whether there were significant differences in the densities of SNPs within the intergenic, intronic or exonic subsets at three different comparative stringencies. However, comparisons between the SNP densities for the exonic and intergenic subsets demonstrate significant differences in the SNP distribution between these subsets (Table 3 and illustrated in Figure 3).

**Table 3:** Mean chromosomal SNP density within the subsets of the conserved genome, at 70% stringency, and statistical comparison of means

	Means			Anova p-value		
**Species**	**E**	**I**	**IG**	**I v E**	**IG v E**	**IG v I**

Pt	2.49	2.50	2.72	0.9022	0.0152	0.0158

Rm	2.49	2.50	2.71	0.8698	0.0177	0.0190

Cf	2.43	2.69	2.69	0.8739	0.0144	0.0160

Mm	2.23	2.23	2.40	0.9491	0.0227	0.0218

Rn	2.23	2.24	2.42	0.8837	0.0176	0.0214

Md	2.10	2.13	2.37	0.6664	0.0061	0.0141

Gg	1.94	1.94	2.26	0.9396	0.0026	0.0042

Xt	1.79	1.75	2.17	0.5955	0.0083	0.0050

Dr	1.80	1.94	2.36	0.1258	0.0002	0.0085

E = conserved Exonic, I = conserved Intronic, IG = conserved Intergenic.

These data suggest that, despite selecting on the basis of conservation, conserved intergenic and conserved exonic sequences appear to be subjected to different strengths of selective pressure within the human population. We considered the possibility that the differences observed in SNP density between the conserved exonic set and the conserved intergenic set reflects differences in the functional mechanism of these sets. The exonic sequences are functionally dependent on a mechanism involving the three base pair codon usage required for coding proteins, whilst intergenic regions and intronic regions are non-coding. Interestingly, comparison of the exonic and intronic subset by ANOVA analysis (Table 3 and illustrated in Figure 3) shows that their SNP densities are not significantly different at any depth of conservation. This observation indicates that intronic and exonic regions in the conserved human genome have been under similar strengths of selective pressure within the human population and have accrued SNPs at very similar rates despite differences in their functionalities.

By contrast, the SNP density within the conserved intergenic subset is consistently higher than in either the conserved coding or intronic subsets irrespective of the species under comparison (see Table 3 and Figure 3). Taking human-chicken conserved regions as an example, the intergenic conserved regions common to chicken and human have an approximately 16% higher SNP density in the human genome than conserved intronic regions conserved to the same degree despite both subsets representing non-coding DNA.

## Discussion

A number of large scale bioinformatic studies have previously explored SNP densities throughout the human genome [[16,24], and [25]]. However, these studies did not compare SNP densities in portions of the genome that had been conserved. The current study has used a whole genome analysis approach using recently published datasets to compare SNP densities within mechanistically different portions of the conserved human genome using a novel set theory approach. We have shown that SNP density decreases throughout the entire conserved genome with increased evolutionary conservation and that the conserved exonic portion contains a significantly reduced SNP density compared to the conserved intergenic region. These results confirm previous hypotheses on a pan-genomic basis and demonstrate that our novel approach is entirely valid. However, the most exciting and novel discovery of the current study is that the intronic portion of the conserved human genome has been subjected to almost exactly the same degree of selective pressure as the exonic component. From a human evolutionary perspective the similarity of selective pressures on the conserved intronic and exonic regions make sense if we consider that, in addition to being properly translated, the primary transcripts also require to be properly spliced. Thus, it has been known for some time that the primary transcripts of as much as 60% of the genome show high levels of tissue specificity in the way that they are alternatively spliced [26-29]. This level of sophistication requires that robust mechanisms be in place to control these processes and these appear to have conserved to the same degrees as exonic sequence and demonstrate identical levels of selective pressure [30]. These observations contradict the previously perceived view of intronic sequences as consisting largely of "junk" DNA, and place the conserved intronic portion of the human genome on a similar level of functional importance in the human population as the conserved exonic genome. Indeed, recent studies have recognised the conservation of splicing regulatory motifs during evolution [31]. The current study supports these conclusions and further suggests that, in addition to being highly conserved, splicing signals within introns have been subjected to proportionately higher selective constraint than intergenic regions within the human population.

The current study also poses a fascinating contradiction. Before the start of this study we predicted that subsets of the genome that had been conserved to the same degree must contain similar densities of SNPs. However, we demonstrate that SNP density between the three subsets analysed is not the same and the intergenic subset contains significantly higher SNP density to either the intronic or exonic subset. This fascinating observation suggests that the variation seen within extant human populations has been selected for in a different way to the variation that has driven vertebrate evolution.

We suggest two alternative hypotheses that might explain why the conserved human intergenic genome contains a higher SNP density than either the exonic or intronic genome. The first hypothesis is that the majority of the sequence within the conserved intergenic genome consists of "junk"; DNA that plays little or no functional role and has been conserved by chance. However, a number of recent studies demonstrating the important role played by conserved intergenic sequence in gene regulation argue against this hypothesis [32]. A second possibility is that the functional mechanisms controlled by the intergenic subset are much more plastic than those of the exonic and intronic subset. Therefore, adaptation to changing selective pressures might be addressed more rapidly and efficiently as a result of regulatory plasticity within the intergenic genome than through possibly deleterious changes in exonic/intronic sequence. Thus, mutations of functional exonic or intronic sequences would be selected against as they are more likely to reduce the fitness of the individual. However, adaptive change would be more likely to be regulatory as the "regulatory genome" mostly represented within the intergenic genome appears to be much more plastic and mutations occurring in this part of the genome can be supported. This second explanation goes some way to supporting the case made by those individuals who believe that phenotypic diversity is primarily driven by non-coding regulatory changes [3].

## Conclusions

The present study suggests the presence of a selective linkage between the exonic and intergenic subsets and ascribes increased significance to the role of introns in human health. In addition, the identification of increased plasticity within the conserved intergenic subset suggests an important role for this subset in the adaptation and diversification of the human population.

## Authors' contributions

SD undertook the design, computer analysis and statistical analysis of the study. AS and AM conceived of the study, and participated in its design and coordination. All authors read and approved the final manuscript.
